# The Workhouse Swill-Tub

**Published:** 1897-04-24

**Authors:** 


### The Workhouse Swill-Tub.

The orders of the Local Government Board in regard to the supply of bread to the inmates of workhouses have always been a source of trouble to those Guardians who are anxious to do their best for the old and feeble folk entrusted to their care. These orders require that each pauper shall receive a given quantity of bread, and also demand that none which has once been served shall be used as food in any form. Hence, when the toothless paupers have munched up what they can there is still plenty for the swill-tub, and the pigs grow fat. In course of time the old people's dietary in certain workhouses has been much improved, at any rate so far as concerns variety, and could be improved more still but for the fact that whatever else is supplied no corresponding diminution can be made in the quantity of bread which day by day must be given out to every pauper.

For some time back it has been the custom at the Chorlton Union, Manchester, to serve only half the authorised quantity of bread, leaving the other half accessible to the inmates, so that they may help themselves to it if they require more. What is left over is then used for making puddings. In practice it has been shown that there is a saving of about 40 per cent. The Hampstead Guardians now wish to do the same, but the Local Government Board are tied by their rules. At a recent meeting of the Guardians the master of the workhouse reported that after three days' trial of the plan of serving only half the authorised weight of bread at the commencement of dinner and the remainder to those who desired it a saving of 24lbs. was effected out of the 56lbs. ordered by the Local Government Board to be supplied, and we are glad to hear that Mr. Chaplin, on being questioned on the matter, has expressed his willingness to receive representations from Guardians on the subject; this is something.

We wish, however, to state definitely, that while we think that the Local Government Board ought to feel at liberty to grant permission for such a variation in the dietary as we speak of in such workhouses as they may approve of, we should strongly deprecate any relaxation of the rule that each pauper is to receive his full allowance of bread unless it is accompanied with some guarantee that what he loses in his loaf will be made up to him in some other equally nourishing form. The fact is that the time has not yet come when the Guardians as a class can be trusted to tamper with the pauper's diet. We thankfully admit that a very considerable number of Boards of Guardians now recognise to the full the important and honorable position which they occupy in the economy of the country, take a pride in their work, and derive an honest pleasure in securing comfort and some amount of happiness for the old and sick who are in their charge. We also know that all the country over, even on the most backward boards, there is a growing minority of good men, and good women also, who, in the face of much opposition and even abuse, are earnestly striving to improve the lot of the aged poor. But the opposition which they meet with is too obvious, the old leaven is too strong, the subservience of many Guardians to the master is too great, to make us dare to ask that as a broad principle it shall be left to the Guardians to decide how much the old people shall have to eat.

A daily loaf is a fearfully monotonous thing no doubt, but in many a country workhouse the order of the Local Government Board, which compels it to be given, is the pauper's charter.

